# Unconventional Pathways of Protein Secretion: Mammals *vs*. Plants

**DOI:** 10.3389/fcell.2022.895853

**Published:** 2022-04-28

**Authors:** Elisa Maricchiolo, Eleonora Panfili, Andrea Pompa, Francesca De Marchis, Michele Bellucci, Maria Teresa Pallotta

**Affiliations:** ^1^ Section of Biological and Biotechnological Sciences, Department of Biomolecular Sciences, University of Urbino Carlo Bo, Urbino, Italy; ^2^ Section of Pharmacology, Department of Medicine and Surgery, University of Perugia, Perugia, Italy; ^3^ Institute of Biosciences and Bioresources, National Research Council of Italy, Perugia, Italy

**Keywords:** extracellular vesicles, vacuole, extracellular space, cell signaling, unconventional protein secretion

## Abstract

In eukaryotes, many proteins contain an N-terminal signal peptide that allows their translocation into the endoplasmic reticulum followed by secretion outside the cell according to the classical secretory system. However, an increasing number of secreted proteins lacking the signal peptide sequence are emerging. These proteins, secreted in several alternative ways collectively known as unconventional protein secretion (UPS) pathways, exert extracellular functions including cell signaling, immune modulation, as well as moonlighting activities different from their well-described intracellular functions. Pathways for UPS include direct transfer across the plasma membrane, secretion from endosomal/multivesicular body-related components, release within plasma membrane-derived microvesicles, or use of elements of autophagy. In this review we describe the mammals and plants UPS pathways identified so far highlighting commonalities and differences.

## 1 Introduction

Eukaryotic cells secrete soluble and membrane proteins during organism development or after induction by different types of stress. The discovery of protein trafficking describes the classical secretion pathway (Vitale and Denecke, 1999), in which proteins are translocated into the endoplasmic reticulum (ER) by a co-translational mechanism that involves the interaction in the cytosol of an N-terminal signal peptide (SP), or a transmembrane domain, with a signal recognition particle (SRP). SRP directs the protein to an ER-localized SRP receptor which, together with an ER-localized translocon complex (Sec61 complex), initiates the ER translocation (Osborne et al., 2005; Shan and Walter, 2005). Secretory proteins are then transported through the Golgi apparatus, to be sorted and targeted to the extracellular space or to the subsequent endomembrane compartments (plasma membrane, vacuoles in yeast and plants, lysosomes in animals, etc.). In recent years, an increasing number of proteins, either with or without an N-terminal SP (leaderless proteins) have been found to reach their final destinations by alternative pathways that bypass the Golgi, leading to the conclusion that this kind of transport is a very important type of protein traffic inside the cell (Goring and Di Sansebastiano, 2017; Pompa et al., 2017). Such proteins, very well represented in the eukaryotic secretome, reach their destination by an “unconventional” mechanism, of which determinants have not yet been clearly defined. In fact, neither the amino acid structural motifs that direct a protein along an unconventional protein secretion (UPS) pathway nor all the biological mechanisms that determine the UPS pathways and the molecular events involved, have been fully characterized. Another aspect related to the UPS definition is that some mechanisms involved in this process are superimposable with other cellular processes such as autophagy or programmed cell death (PCD). Even if the crosstalk between organelles of different pathways may occur in specific situations, UPS is clearly determined by literature as a distinct pathway from the conventional exo–endocytic trafficking which regulates the turnover of plasma membrane proteins (Zhang et al., 2019) and the autophagic route as well (Hu et al., 2020). Currently investigated in many organisms, UPS seems to be related to several physiological processes like immune responses, abiotic stress responses and cell proliferation in normal growth conditions (Pallotta and Nickel, 2020; Balmer and Faso, 2021). In this review, along with a presentation of differences and commonalities between the UPS mechanisms in mammals and plants, we try to summarize the latest research on UPS to combine the molecular mechanisms and the physiological issues of this type of protein transport.

### 1.1 Mechanisms and Physiological Role of Unconventional Protein Secretion in Mammalian Cells

In mammals, many leaderless proteins can be secreted outside the cells through different UPS mechanisms. Such secretion is biologically controlled because these proteins can exploit distinct extracellular functions, like immune modulation or cell signaling, activities different from their intracellular ones (Cohen et al., 2020). Some proteins are directly translocated across the plasma membrane forming pore structures. Generally, these proteins bind lipids, undergo a conformational change facilitated by other proteins, and then pass through the plasma membrane (Stewart et al., 2018). Only in a few cases protein secretion turns out to be mediated by ABC transporters, while a wide range of proteins is taken up into intracellular vesicle intermediates and released upon fusion with the plasma membrane in a free form or into vesicles (Cocozza et al., 2020). Moreover, integral membrane proteins lacking SP are translocated from the ER to the plasma membrane without the passage through the Golgi apparatus (Pallotta and Nickel, 2020).

#### 1.1.1 Type I Pathway: Golgi-Bypass Pathway for Leaderless Proteins

In mammals, UPS type I is a secretory pathway wherein soluble leaderless proteins directly translocate across the plasma membrane. One of the first and most studied proteins that undergo this pathway is Fibroblast Growth Factor 2 (FGF2), which is recruited at the inner plasma membrane leaflet through interaction with the α1-subunit of the Na/K-ATPase (Legrand et al., 2020). This event promotes FGF2 binding to the phosphoinositide PI(4,5)P_2_ and the recruitment of the kinase Tec. FGF2 first oligomerizes, to be then phosphorylated by kinase Tec forming lipidic membrane pores. Lastly, membrane inserted FGF2 oligomers are disassembled at the outer plasma membrane leaflet by membrane proximal heparan sulfate proteoglycans, and FGF2 appears on the cell surface (Legrand et al., 2020; Pallotta and Nickel, 2020).

The UPS mechanism of FGF2 has proved to be relevant also for other functionally different proteins such as Tau and human immunodeficiency virus type 1 transactivator of transcription (HIV-Tat). Like FGF2, the secretory process of these proteins occurs by direct translocation across the plasma membrane and requires both PI(4,5)P_2_ for the binding to the inner leaflet and heparan sulfates for the release from the outside leaflet (Mele et al., 2018; Merezhko et al., 2020). Furthermore, the secretion of HIV-Tat involves the binding to the α1-subunit of the Na/K-ATPase as well (Agostini et al., 2017). The recent case of the protein engrailed-2 homeoprotein (EN2) translocated across the plasma membrane due to its interaction with PI(4,5)P_2_ also suggests that the EN2 secretion may rely on a UPS type I pathway (Amblard et al., 2020).

Interestingly, several aspects of the FGF2 secretion pathway also seem to be relevant for interleukin-1β (IL-1β). IL-1β, an essential cytokine necessary for acute inflammatory responses, is produced in the cytosol as a precursor (pro-IL1β). After the cleavage by caspase-1 into a mature form (mIL-1β), mIL-1β, like FGF2, is targeted to the plasma membrane in a PI(4,5)P_2_-dependent manner and then exits the cell through membrane pores. However, unlike FGF2, mIL-1β does not appear to interact directly with PI(4,5)P_2._ The membrane pores formation that allows mIL-1β passage is triggered by phosphoinositide-dependent oligomerization of the N-terminal domain of the cytosolic protein Gasdermin D, which is generated through proteolytic cleavage by inflammasome-activated caspases (Chan and Schroder, 2020). Moreover, by forming pores in the plasma membrane, the cleaved Gasdermin D ultimately causes cell lysis in a cell death process named pyroptosis (Evavold et al., 2018).

#### 1.1.2 Type II Pathway: ABC Transporter-Based Secretion

In mammalian cells, a few proteins are known to be secreted through the Type II UPS pathway, which allows protein translocation through the plasma membrane *via* ATP-binding cassette (ABC) transporters (Dimou and Nickel, 2018). The first member of the ABCA subfamily, named ABCA1, promotes the secretion of several proteins, such as acetylated apurinic (apyrimidinic) endonuclease-1/redox factor-1 (AcAPE1/Ref-1) (Chen et al., 2021) and macrophage migration–inhibitory factor (MIF) (Sitia and Rubartelli, 2020). Heat shock 70-kDa protein (HSP70), which can be secreted through Type II UPS, appears to enter into endolysosomal vesicles with the aid of ABC transporters spanning the lysosomal membrane and to exit from mammalian cells *via* these vesicles (Cohen et al., 2020). Moreover, HSP70 seems to be capable of mediating a mechanism of type I UPS by itself. In fact, HSP70 associates with lipid membranes and, upon membrane insertion, oligomerizes and forms ion conductance channels. As a result, it mediates the extracellular secretion of different proteins (De Maio and Hightower, 2021).

#### 1.1.3 Type III Pathway: Organelle-Based Translocation and Extracellular Vesicles

Type III UPS pathway involves different types of organelles that are in some cases intracellular vesicle intermediates, especially secretory lysosomes, multivesicular bodies (MVBs) and secretory autophagosomes. Endosomes, autophagosomes, and lysosomes are membrane-bound organelles with their normal cellular functions, but turn out to be secretory organelles after induction by stress signaling pathways (Pallotta and Nickel, 2020).

In mammals, MVBs are crucial components of the endolysosomal system, which leads to endocytosis, recycling, and degradation of different kinds of macromolecules, including proteins. The membrane of MVBs invaginates, captures membrane and cytosolic proteins into vesicles and forms intraluminal vesicles (ILVs). Upon fusion of these compartments with the plasma membrane, proteins are secreted outside the cell into extracellular vesicles (EVs) named exosomes (Cocozza et al., 2020). Exosomes represent one of the major types of EVs (Mathieu et al., 2019) with contents and markers defined by previous studies (Jeppesen et al., 2019). The membrane of classical exosomes contains CD63, CD81, CD9, flotillin -1 and -2, EGFR, integrin beta1 and alpha2, and Na/K-ATPase. Four different complexes named endosomal sorting complexes required for transport 0-III (ESCRT 0-III) control the generation of ILVs, in particular monomers of the ESCRT-III protein Snf7, which polymerize, deform the membrane and allow the vesicles fission (Cohen et al., 2020).

Different types of vesicles can be released directly from the plasma membrane, such as ectosomes, microvesicles, microparticles, large oncosomes and apoptotic bodies (Cocozza et al., 2020).

Being derived from the pinching outwards of the plasma membrane, microvesicles can recruit cytosolic leaderless proteins. Like the blebbing mechanism, firstly the cytoskeleton adjacent to the site of shedding on the plasma membrane is disassembled, then the phosphatidylserine is translocated to the outer leaflet causing the plasma membrane to bulge (Cohen et al., 2020). Functional microvesicles are involved in several physiopathologic conditions, such as inflammation, oxidative stress, and senescence (Hijmans et al., 19852019). These vesicles differ from exosomes not only in formation mechanism, but also in size and molecular markers. Being heterogeneous, bigger than exosomes (50–1,000 nm in diameter whereas exosomes are between 30 and 150 nm (van Niel et al., 2018)), they contain glucose-regulated protein 94 (GRP94, also known as GP96), tumor susceptibility gene 101 (TSG101), annexin A1 and ADP-ribosylation factor 6 (ARF6) as markers (Jeppesen et al., 2019).

An interesting secretion route involves secretory lysosomes. Lysosomes can generate not only protein degradation, but also protein secretion. For this purpose, these vesicles release proteins by fusing with the plasma membrane and liberating their contents in the extracellular space. Studies on fatty acid-binding protein FABP4 show how a protein is secreted by this mechanism (Villeneuve et al., 2018).

Another way of leaderless protein release outside the cell is mediated by the so-called misfolding-associated protein secretion (MAPS) pathway. Cytotoxic polypeptides, such as alpha-synuclein, Tau and other cytosolic misfolded proteins, are delivered to late endosomes, which then fuse to the plasma membrane, releasing their contents (Sitia and Rubartelli, 2020).

Structures involved in autophagy are also critical for UPS of leaderless proteins, such as autophagosomes and amphisomes. Autophagosomes are double-membrane organelles formed under starvation and exogenous stresses to break down cellular components, but they are also constitutively formed to maintain the turnover of self-components. Moreover, autophagy can selectively degrade harmful substances that cannot be digested by other pathways such as the proteasomal degradation pathway (Kawabata and Yoshimori, 2020). Being capable of capturing other organelles and large areas of cytoplasm, autophagosomes deliver the materials to lysosomes or MVBs or the extracellular space for recycling, degradation or secretion of the cargo (Pallotta and Nickel, 2020). In particular, when autophagosomes fuse with MVBs, structures called amphisomes are formed, which can later fuse with the plasma membrane and deliver cargo to the external environment as a UPS mechanism (Cohen et al., 2020). An example of protein secreted by this mechanism is histone H3 (Jeppesen et al., 2019), while IL-1β can be released outside the cells through autophagosomes. Cytokine IL-1β can be released by either pyroptosis and pore formation (Type I UPS) or autophagy-mediated UPS mechanism (Type III UPS). Recent studies have revealed the mechanism by which IL-1β and other leaderless cargoes enter into the lumen of intracellular vesicle intermediates, in order to be secreted by the type III UPS pathway (Zhang et al., 2020). The transmembrane p24 trafficking protein 10 (TMED10) plays a crucial role in vesicle entry, as well as the secretion of many leaderless cargoes, like IL-1β. The unfolded form of this cytokine is bound to the cytoplasmic chaperone heat shock 90-kDa protein (HSP90A), which directs the protein to TMED10 localized in the ER-Golgi intermediate compartment (ERGIC). TMED serves as a protein channel and directs the entry of cargoes into this structure (Zhang et al., 2020). Besides, components of the early secretory pathway named Golgi reassembly and stacking proteins (GRASP, in mammals GRASP55 and GRASP65), are involved in the biogenesis of the vesicle intermediates, turning out to be important for IL-1β secretion (Chiritoiu et al., 2019).

#### 1.1.4 Type IV Pathway: Bypassing the Golgi With SP/Transmembrane Domain-Containing Proteins

In mammals, type IV UPS is a pathway where integral membrane proteins translocated into the ER reach the plasma membrane bypassing the Golgi apparatus (therefore defined as Golgi-bypass). It is mostly associated with cellular stress signals generated during nutrient starvation, mechanical stress and ER stress. Indeed, proteins specialized in recognizing misfolded proteins and implicated in ER stress response, like IRE1, GRASPs, heat shock proteins as well as their cofactors and molecular chaperones, take part in the Golgi-bypass of different cargo proteins (Gee et al., 2018).

Well-known examples of transmembrane proteins that undergo the type IV UPS pathway are pendrin and cystic fibrosis transmembrane conductance regulator (CFTR). Disease-causing mutations of both CFTR and pendrin lead to, proteins misfolding and retention in the ER. Studies have demonstrated that under blocked ER-to-Golgi transport or ER stress conditions, immature core-glycosylated CFTR and pendrin can reach the plasma membrane *via* the Golgi-bypass UPS pathway and retain their anion transporting activity. The basic mechanisms by which these two proteins reach the cell membrane *via* UPS appear to be similar, both enhanced by IRE1α kinase pathway activation (Park et al., 2020). However, some key molecules controlling the UPS of these two membrane proteins are not identical. For example, GRASP55 is required for the UPS of CFTR, whereas the HSP70 co-chaperone DNAJC14 is involved in the UPS of pendrin (Gee et al., 2018; Zhang and Wang, 2020). Furthermore, vesicular components related to autophagosome formation are involved in UPS. For instance, only knockdown of components in the autophagosome formation (ATG1, ATG5, ATG7, and ATG8), but not that of vacuole fusion (Vamp7), inhibits unconventional surface trafficking of the mutated form of CTFR (ΔF508-CFTR) (Gee et al., 2018). Moreover, secretory autophagy machinery and vesicular trafficking components have been demonstrated to take part in the secretory pathway of high mobility group box 1 (HMGB1), a leaderless protein whose unconventional secretion mechanism has recently been clarified (Kim et al., 2021). In particular, the machinery of HMGB1 secretion is mediated by Golgi reassembly stacking protein 2 (GORASP2), secretion associated Ras-related GTPase 1A (SAR1A^T39N^), ADP ribosylation factor 1 (ARF1^Q71L^) and MVBs formation (Kim et al., 2021). However, important questions regarding the mechanism of autophagy-mediated UPS of transmembrane proteins remain to be elucidated (Noh et al., 2018).

It should be noted that certain cargoes can enter different types of UPS pathways based on the physiological context. Indeed, some proteins that undergo the type I pathway can reach the extracellular space through other UPS mechanisms as well. Typical examples are Tau undergoing both type I UPS and UPS by EVs, and IL-1β going through both type I and type III UPS (Pallotta and Nickel, 2020).

### 2.2 Mechanisms and Physiological Role of Unconventional Protein Secretion in Plants

Numerous review articles have already described UPS in plants (De Marchis et al., 2013a; Davis et al., 2016; Robinson et al., 2016) or compared conventional protein secretion with UPS in plants (Goring and Di Sansebastiano, 2017; Wang et al., 2017). In one of these papers, (Ding et al., 2014) the authors have suggested a classification for the different types of UPS existing in plants: type I, a Golgi-bypass pathway for SP-lacking polypeptides, type II, a secretion route mediated by the vacuole, or (type III) mediated by MVBs, or (type IV) mediated by an exocyst-positive organelle (EXPO). Unfortunately, other authors have denominated UPS types in animals and yeast as type I-IV, and at least in two cases, very different UPS mechanisms share the same name. For example, the type IV pathway in mammalian cells involves SP- and/or transmembrane domain-containing proteins which are translated in the ER and then targeted to the plasma membrane without passing through the Golgi (Rabouille, 2017). Conversely, the type IV pathway in plant cells corresponds to a secretory pathway mediated by EXPO, a double-membrane-bound organelle that fuses with the plasma membrane and releases leaderless cytosolic proteins (Ding et al., 2014; Wang et al., 2020). Therefore, we describe in this paper how the UPS plant classification system should be revised in comparison to the UPS general categories identified by Pallotta and Nickel (Pallotta and Nickel, 2020).

#### 2.2.1 Type I Pathway: Golgi-Bypass Pathway for Leaderless Proteins

Both mammalian and plant cells use this UPS route, and many leaderless secretory proteins have been described in the plant secretome (Agrawal et al., 2010; Krause et al., 2013), but in plants, there are only a few published examples of leaderless proteins secreted in the apoplast bypassing the Golgi apparatus. One involves a leaderless heterologous protein of bacterial origin, hygromycin phosphotransferase (HYGR), which is secreted, when expressed in transgenic Arabidopsis plants, from the cytosol to the apoplast, i.e the plant extracellular space, bypassing the Golgi (Zhang et al., 2011). With this aim, brefeldin A (BFA), an inhibitor of protein traffic through the Golgi apparatus caused by deregulated fusion of the ER with the Golgi cisternae, has been used. BFA treatment does not inhibit HYGR secretion (Zhang et al., 2011), nor does it impede the secretion of another protein, mannitol dehydrogenase (MTD) (Cheng et al., 2009). MTD, the only other example, converts mannitol to mannose and it is localized in the cell (cytoplasm, nucleus, etc.) but secreted into the apoplast after treatment with salicylic acid, an endogenous inducer of plant defense responses (Cheng et al., 2009). MTD secretion may represent part of a plant defense mechanism against mannitol-secreting fungal pathogens, and a very preliminary effort has recently been made to identify the cytoplasmic components of the MTD secretory machinery following salicylic acid treatment (Ho et al., 2022). Indeed, no information about the translocation mechanisms of HYGR and MTD is available yet.

#### 2.2.2 Type II Pathway: ABC Transporter-Based Secretion

No plant-secreted protein seems to follow this route involving lipidated cargoes and being mediated by ABC transporters (Dimou and Nickel, 2018).

#### 2.2.3 Type III Pathway: Organelle-Based Translocation and Extracellular Vesicles

Through similar mechanisms used by mammalian cells, the endomembrane trafficking system in plants is tightly linked to cellular stresses in order to rapidly adapt the cellular processes to the new physiological conditions (Wang et al., 2020). The vacuole is the largest membrane-bounded compartment in plant cells with multiple functions essential for plant growth and development, and some of these functions, like cellular waste degradation, are similar to those of lysosomes. In case of a pathogen attack, vacuoles can turn into secretory organelles and fuse with the plasma membrane at pathogen entry sites releasing antibacterial proteins like aleurain, aspartyl protease, and carboxypeptidase Y (Hatsugai et al., 2009). These hydrolytic enzymes enter the ER due to their N-terminal SP and then traffic along the conventional protein secretion pathway to reach the vacuole where they normally degrade cellular proteins. Their induced release into the apoplast carries out both antibacterial activity and cell death-inducing activity, leading to PCD as a defense strategy developed by plants for lack of immune cells (Ruano and Scheuring, 2020).

In plants, another UPS route should be comprised of the type III pathway because it is organelle-mediated and the secreted proteins are released as part of vesicles: UPS mediated by EVs. Plant EVs can be secreted from either exocyst-positive organelles (EXPOs) or MVBs.

EXPOs, double-membrane organelles from 500 to 800 nm in diameter (similar to autophagosomes), are Exo70E2-positive structures because immunolabelling studies have shown the exocyst subunit Exo70E2 co-localized with them (Wang et al., 2010). EXPOs deliver cargo-containing vesicles into the apoplast by fusion of the outer membrane with the PM, while the inner boundary membrane is subjected to degradation (Ding et al., 2012). EXPO characteristics have recently been revised (Cui et al., 2020), so here we only underline that EXPOs seem to be involved in releasing exosomes containing leaderless proteins with a role in growth regulation and plant cell wall remodeling. De Caroli and colleagues (De Caroli et al., 2021) have shown that two out of three xyloglucan endotransglucosylase/hydrolases (XTHs) involved in cell wall assembly are targeted to the cell wall and plasma membrane through a conventional protein secretion pathway. Conversely, the other leaderless protein (XTH29), released in the apoplast by a UPS route mediated by EXPOs, appears to be upregulated in response to abiotic stresses.

Plant MVBs (alternatively named prevacuolar compartments or late endosomes) are organelles of conventional secretion pathway mediating the transport from the Golgi to vacuoles (Hu et al., 2020), but MVBs can also participate in UPS pathways by fusing with the plasma membrane to release their ILVs to the apoplast. These ILVs, referred to as exosomes, take part in intercellular communication and carry small RNAs and proteins (Cai et al., 2018). Exosomes belong to an EVs subpopulation of 30–150 nm in diameter, isolated by differential ultracentrifugation (Rutter and Innes, 2020; Kim, 2021) and enriched in membrane proteins used as biomarkers like the syntaxin AtSYP121/PENETRATION1 (PEN1) and the tetraspanin (TET) 8 and TET9 (Rutter and Innes, 2017; Cai et al., 2018). Transmission electron microscopy images of MVBs and exosomes have been shown in plant leaves (An et al., 2006; Liu et al., 2020) and stigmatic papillae (Goring, 2017), moreover, exosomes have been isolated from external fluids of leaves (Rutter and Innes, 2017), pollen grains (Prado et al., 2014) and seeds (Pinedo et al., 2012). However, there is limited experimental evidence of MVB-plasma membrane fusion resulted in the release of exosomes into the apoplast (Movahed et al., 2019). Since the secretion of plant exosomes/EVs is enhanced in response to pathogen infection (Hansen and Nielsen, 2017; Rybak and Robatzek, 2019), and proteomic analyses of exosomes/EVs isolated from plant tissues have identified enrichment of proteins involved in cell wall remodeling enzymes and defense/stress-related proteins (Regente et al., 2017; Rutter and Innes, 2017), it is widely accepted that plant exosomes/EVs perform a function in plant growth and development (de la Canal and Pinedo, 2018), including regulation of plant-microbe interactions (Ivanov et al., 2019; Roth et al., 2019; Cui et al., 2020). Nevertheless, many questions remain to be answered, like which cargo proteins are involved and how they are loaded in plant exosomes.

#### 2.2.4 Type IV Pathway: Bypassing the Golgi With SP/Transmembrane Domain-Containing Proteins

Integral membrane proteins, synthesized in the ER and bypassing the Golgi during their journey to the plasma membrane, are comprised in this route. In plants, there is no solid result able to demonstrate protein traveling from the ER to the plasma membrane or the apoplast bypassing the Golgi apparatus. Alternatively, there are many examples of proteins directly delivered from the ER to the vacuole, which have extensively been reviewed (Pedrazzini et al., 2016; Bellucci et al., 2017). Both soluble and membrane proteins with ER-targeting signals can traffic to the vacuole bypassing the Golgi, including proteins aggregated in large polymers (ER bodies), which are stored either in seed tissues to allow seed germination or in vegetative tissues to join the plant defense against abiotic stresses. Examples of such ER bodies are the precursor-accumulating vesicles (PACs) and the ER bodies described in plants of the *Brassicales* order. PACs are ER-derived spherical bodies that accumulate storage proteins and, after being released in the cytoplasm, fuse with the protein storage vacuoles (Hara-Nishimura et al., 1998). In *Arabidopsis thaliana* (Brassicaceae) seedlings, ER bodies accumulate mainly proteases and fuse to the vacuoles in presence of salt stress, thus assisting the cell death under stress conditions (Hayashi et al., 2001).

Heterologous expression of soluble glycoproteins, like human lysosomal alpha-mannosidase (MAN2B1) and mouse IgG1 14D9, demonstrates that these proteins directly reach the vacuole after translocation into the ER (De Marchis et al., 2013b; Ocampo et al., 2016). It is not clear how they are delivered to the vacuole and if glycosylation is relevant for their trafficking. Interestingly, in the case of cardosins, plant vacuolar aspartic proteinases, when the C-terminal vacuolar sorting domain (VSD) is artificially removed, a second domain named plant-specific insert (PSI) acts as a VSD in specific conditions or developmental stages. In the species artichoke (*Cynara Cardunculus*) and soybean (*Glycine max*), the glycosylation status of the PSI domain seems to play an important role in determining if the cardosin should go through or bypass the Golgi in their route to the vacuole (Vieira et al., 2019). As regards membrane proteins, several proteins located in the vacuole membrane like calcineurin B-like (CBL) 6, soluble N-ethylmaleimide sensitive factor attachment protein receptor (SNARE) VAM3 and α-TIP, traffic through this UPS pathway (Di Sansebastiano et al., 2017).

## 2 Discussion

**FIGURE 1:**
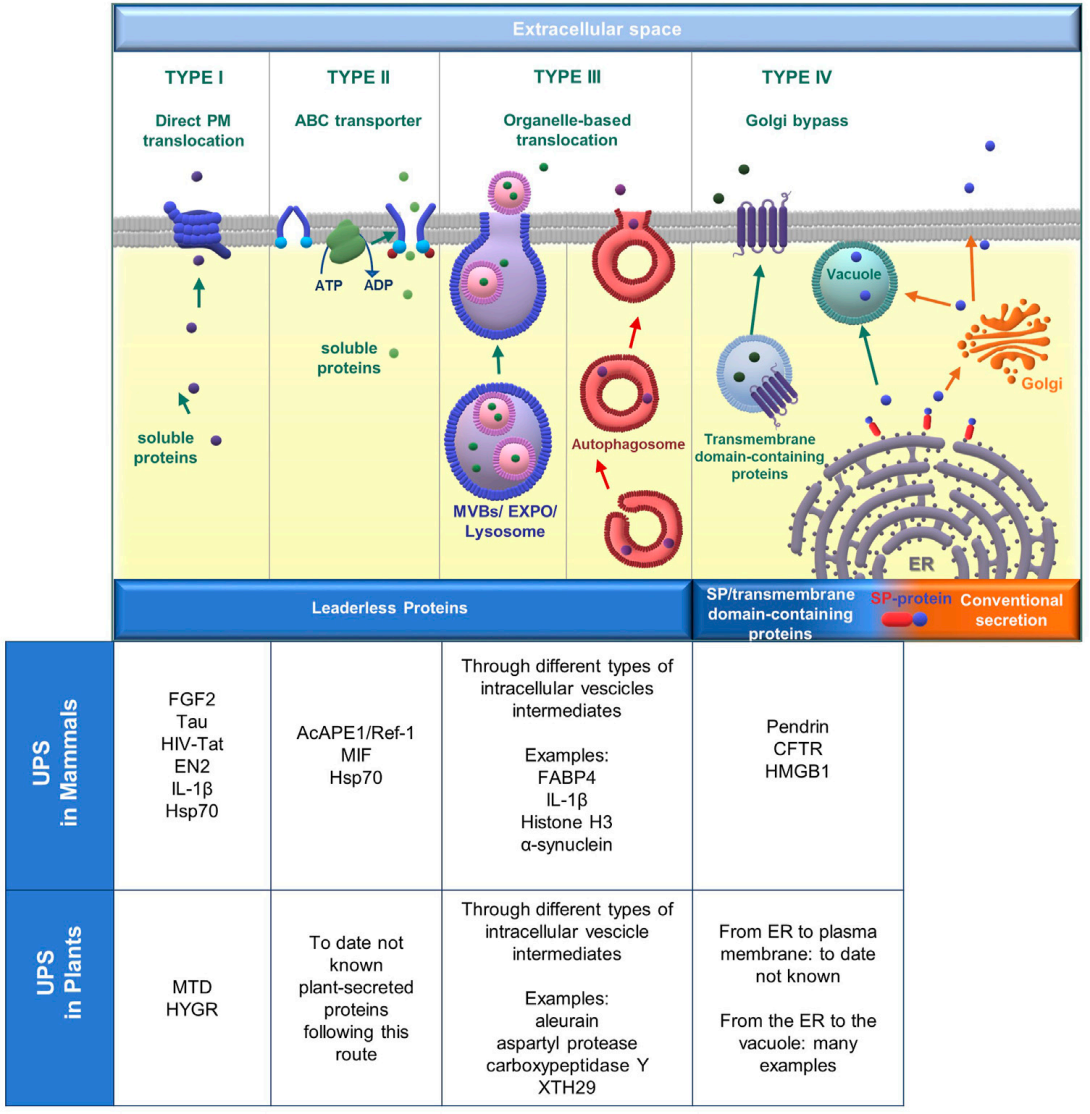
Overview of unconventional protein secretion (UPS) pathways in mammals and plants, with a description of proteins following different UPS routes. From left to right: UPS type I, UPS type II, and UPS type III are used by leaderless proteins which employ different methods to go through the plasma membrane (PM). In type III autophagosomes are inserted to represent the crosstalk between UPS and autophagy (see Paragraph 1.1.3). The scheme of UPS type IV represents the destiny of SP/transmembrane domain-containing proteins translocated in the ER, the next journey to the plasma membrane in mammals, and the direct delivery to the vacuole in plants. However, such proteins normally traffic along the conventional secretory pathway and transit through the Golgi apparatus.

We are firmly convinced that a unique classification system in mammals and plants for the UPS pathways based on their different molecular mechanisms should be an important prerequisite for biological research to avoid confusion. After all, similar motivations have driven the community to conceive biological classification systems (Marakeby et al., 2014). A re-classification of the UPS pathways can especially benefit the plant scientific community because in plants the understanding of UPS mechanisms is still very restricted and the assignment of a particular protein transport route to a type of UPS pathway can arise debate (Pompa et al., 2017). This is the case of EXPO organelles, and in fact for some authors the question if these organelles should be considered part of an autophagic transport to the vacuole rather than part of a UPS pathway that releases leaderless proteins in the apoplast, is still in discussion (Kulich et al., 2013; Lin et al., 2015). However, with the scientific data available about the UPS pathways, abundant in mammals and few in plants, we’ve succeeded in finding some common categories/types (Figure 1). For example, there are many similarities between animals and plants in UPS type III, where organelles normally involved in the endomembrane trafficking system of the classical secretion pathway (vacuoles in plants and lysosomes in animals) become UPS organelles, but there are also differences due to specific membrane-bound organelles like the EXPOs in plants. Moreover, specialized UPS organelles, such as the yeast cup-shaped membranes (CUPS), are present neither in animals nor in plants (Bruns et al., 2011). It has been difficult for us to distinguish proteins secreted by UPS routes from those secreted by autophagy or PCD mechanisms because interaction takes place between UPS and autophagy/PCD. Conversely, we have unanimously decided to exclude from this review plasmodesmata and tunnelling nanotubes which are types of cell-to-cell transport based on intercellular channels (Knox and Benitez-Alfonso, 2014). By writing this review, we have realized that knowledge is still very limited of the molecular machineries involved in the secretion of proteins by unconventional pathways both in animals and in plants. The UPS topic will become more and more important in the coming years and an increasing understanding of these secretion mechanisms will provide unique opportunities for applied biology.
